# The complete mitochondrial genome of Huainan partridge chicken (*Gallus gallus*)

**DOI:** 10.1080/23802359.2020.1847616

**Published:** 2021-01-19

**Authors:** Shu-ying Peng, Ming-xin Lu, Min Wang, Ling Wang, Cheng-qiao Wang, Jing-ru Wang, Yun-fang Zhang, Hai-jun Zhang, Jun Li

**Affiliations:** Human and Animal Genetics Laboratory, School of Life Science, Huaibei Normal University, Huaibei, Anhui, P.R. China

**Keywords:** Gallus, Huainan Partridge chicken, mitogenome, phylogenetics

## Abstract

Huainan Partridge chicken is one of the indigenous chicken breeds in China. In this study, the first complete mitochondrial DNA (mtDNA) sequence of Huainan Partridge chicken had been obtained using PCR amplification, sequencing and assembling. The mitogenome of Huainan Partridge chicken is 16785 bp in length, including a control region (D-loop), 13 protein-coding genes, 22 transfer genes and 2 ribosomal genes. The base composition of the complete mtDNA sequence is 30.27% for A, 23.73% for T, 13.50%for G, 32.50% for C. This study will provide reference for the phylogenetic analysis of Huainan Partridge chicken.

Huainan Partridge chicken is an excellent native chicken breed in Anhui Province, China. This breed that is known for good meat quality and egg-laying performance has been included in the ‘Convention on Biological Diversity’. Huainan Partridge chicken is mainly distributed in the south of the Huaihe River (Chen et al, 2015). This chicken has a unique feather color character, including green shank, green beak, the whole body of rooster is red feather, and that of hen is spotted-brown feather (Geng et al. 1994; Li et al. 2011).

In order to explore the molecular evolution of Huainan Partridge chicken, we investigated the complete sequence of Huainan Partridge chicken mitogenome and its phylogenetic relationship with other indigenous chicken in China for the first time. In this study, Huainan Partridge chicken was obtained from a local conservation farm in Huainan city (N 31°54′8″–33°00′26″, E116°21′5″–117°12′30″), Anhui Province, China. The whole blood samples were drawn from the wing vein. The blood specimen was stored at −80 °C in the Museum of the Human and Animal Genetics Laboratory, School of Life Science, Huaibei Normal University, China (Voucher No. HPCb20191001). Total genomic DNA was extracted from these blood samples by using the TIANamp Genomic DNA Exaction Kit (TIANGEN Biotech Co.Ltd, Beijing, China) according to its instruction manual. The mitogenome was amplified with 10 pairs primers designed according to the mitogenome sequence of Gallus (NC_007236), and PCR products were sequenced by BGI Biotech Co.Ltd (Shanghai, China). Mitogenome sequences were assembled using Seqman software (DNASTAT Inc., Madison, WI, USA) and annotated using MITOS WebServer (http://mitos.bioinf.uni-leipzig.de) (Bernt et al. 2013). The Neighbor-joining (NJ) phylogenetic tree was constructed using Mega 7.0 (Kimura 1980; Kumar et al. 2016) with 1000 bootstrap replicates.

The complete mitogenome of Huainan Partridge chicken was 16,785 bp in length (GenBank accession number MN972459). The overall base composition of mitogenome was 30.27% A, 23.73% T, 32.50% C and 13.50% G and biased A + T (54.00%). It consisted of 37 mitochondrial genes, including 13 protein-coding genes (PCGs), 22 transfer RNAs, 2 rRNA (12S rRNA and 16S rRNA) and a non-coding control region (D-loop region). The arrangement of all genes and transcriptional direction were identical with those of typical Gallus (Desjardins and Morais 1990). The mitochondrial genome was very closely arranged with gene overlaps. The total length of gene overlaps was 32 bp in 8 different locations. The total length of intergenic spaces was 45 bp in 15 different locations ranging from 1 bp to 9 bp, with the largest interval between tRNA^Leu^-ND1 (9 bp). Except for the ND6 gene and eight tRNA genes (tRNA^Gln^, tRNA^Ala^, tRNA^Asn^, tRNA^Cys^, tRNA^Tyr^, tRNA^Ser^, tRNA^Pro^ and tRNA^Glu^), all mitogenome-genes were encoded on the heavy strand. The PCGs initiation codons were all ATG, except for COI that begins with GTG. The termination codons for COII, ATPase6, ATPase8, ND1, ND3, ND4L, ND5, Cytb and ND6 genes were TAA, the termination codon for ND2 was TAG, the termination codon for COI gene was AGG, and the termination codon for ND4 and COIII genes was incomplete termination codon ‘T––’ that was the 5′ terminal of the adjacent genes. The incomplete ‘T––’ was supposed to become TAA via posttranscriptional polyadenylation (Anderson et al. 1981). In addition, a cytosine insertion existed in the ND3 gene. An extra base at position 10,965 was discovered, but the reading frame was presumably maintained by translational frameshift of RNA. This phenomenon should be similar to the single nucleotide frameshift insertions in many birds (Mindell et al. 1998). The chicken mitochondrial genome contained 12 S rRNA and 16 S rRNA. Two ribosomal RNAs were located between the tRNA^Phe^ and tRNA^Leu^ genes, and separated by the tRNA^Val^ gene. The lengths of 12 S rRNA and 16 S rRNA genes were 976 bp and 1 624 bp, respectively. The deduced 22 tRNA genes in the chicken mitochondrial genome were distributed among rRNA and protein-coding genes, ranging from 66 ∼ 76 nt. The D-loop region is a 1232 bp long sequence between tRNA^Phe^ and tRNA^Glu^, accounting for 7.3% of the whole mitogenome.

1. The neighbor-joining tree was constructed based on the complete mitogenomes of Huainan Partridge chicken and other 20 indigenous chicken breeds using Mega 7.0 (Kimura 1980; Felsenstein 1985; Saitou et al. 1987; Kumar et al. 2016) with 1000 bootstrap replicates to elucidate the phylogenetic position of Huainan Partridge chicken. The phylogenetic tree showed that Huainan Partridge chicken is close with Guangxi Partridge chicken and Xuefeng chicken. These three chickens with Rugao chicken, Tulufan chicken and Xianju chicken form a monophyletic clade (Figure 1). This study will provide a reference for phylogenetic analysis of Huainan Partridge chicken, and contribute to the protection and breeding of indigenous chicken.

**Figure 1.**
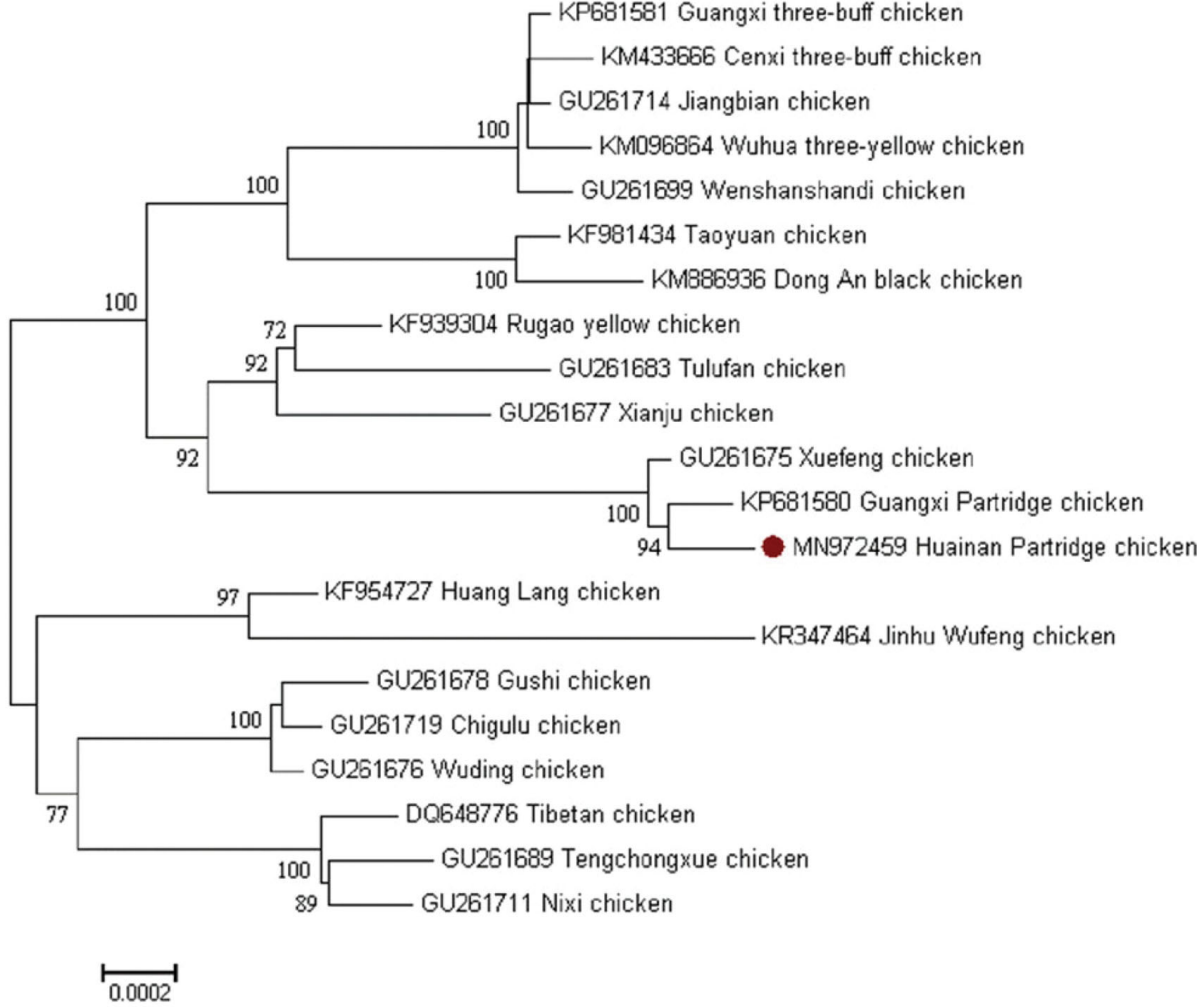
Neighbor-joining tree based on the complete mitogenome of 21 indigenous chicken breeds. GenBank accession number are given before the species name.

## Data Availability

The complete mitochondrial genome of Huainan Partridge chicken (Gallus gallus) is openly available in GenBank at http://www.ncbi.nlm.nih.gov/genbank with accession number MN972459.
